# DNA‐Grafted Hyaluronic Acid System with Enhanced Injectability and Biostability for Photo‐Controlled Osteoarthritis Gene Therapy

**DOI:** 10.1002/advs.202004793

**Published:** 2021-03-01

**Authors:** Zhijie Chen, Feng Zhang, Hongbo Zhang, Liang Cheng, Kaizhe Chen, Jieliang Shen, Jin Qi, Lianfu Deng, Chuan He, Hélder A. Santos, Wenguo Cui

**Affiliations:** ^1^ Department of Orthopaedics Shanghai Key Laboratory for Prevention and Treatment of Bone and Joint Diseases Shanghai Institute of Traumatology and Orthopaedics Ruijin Hospital Shanghai Jiao Tong University School of Medicine 197 Ruijin 2nd Road Shanghai 200025 P. R. China; ^2^ Drug Research Program Division of Pharmaceutical Chemistry and Technology Faculty of Pharmacy University of Helsinki Helsinki FI‐00014 Finland; ^3^ Pharmaceutical Sciences Laboratory and Turku Bioscience Centre Åbo Akademi University Turku FI‐20520 Finland; ^4^ Helsinki Institute of Life Science (HiLIFE) University of Helsinki Helsinki FI‐00014 Finland

**Keywords:** anti‐inflammation, DNA grafted hyaluronic acid, enhanced injectability, long‐term bio‐stability, osteoarthritis gene therapy, spherical nucleic acids

## Abstract

Gene therapy is identified as a powerful strategy to overcome the limitations of traditional therapeutics to achieve satisfactory effects. However, various challenges related to the dosage form, delivery method, and, especially, application value, hampered the clinical transition of gene therapy. Here, aiming to regulate the cartilage inflammation and degeneration related abnormal IL‐1*β* mRNA expression in osteoarthritis (OA), the interference oligonucleotides is integrated with the Au nanorods to fabricate the spherical nucleic acids (SNAs), to promote the stability and cell internalization efficiency. Furthermore, the complementary oligonucleotides are grafted onto hyaluronic acid (HA) to obtained DNA‐grafted HA (^DNA^HA) for SNAs delivery by base pairing, resulting in significantly improved injectability and bio‐stability of the system. After loading SNAs, the constructed ^DNA^HA‐SNAs system (HA‐SNAs) performs a reversible NIR‐triggered on‐demand release of SNAs by photo‐thermal induced DNA dehybridization and followed by post‐NIR in situ hybridization. The in vitro and in vivo experiments showed that this system down‐regulated catabolic proteases and up‐regulated anabolic components in cartilage over extended periods of time, to safeguard the chondrocytes against degenerative changes and impede the continual advancement of OA.

## Graphical abstract

A NIR light‐triggered thermal‐assisted gene therapy system is constructed to safeguard the chondrocytes against degenerative changes and impede the continual advancement of osteoarthritis (OA). The interference oligonucleotides with the Au nanorods to fabricate the spherical nucleic acids is integrated to promote the stability and cell internalization efficiency, which regulated the cartilage inflammation and degeneration related abnormal IL‐1*β* mRNA expression in OA.

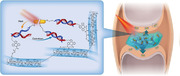

## 1 Introduction

As one of the most common degenerative diseases, osteoarthritis (OA) has become a public health challenge in the current aging society, with worldwide estimates indicating that 250 million people are affected nowadays.^[^ ^1^ ^]^ OA has not only been deemed as lubrication deficiency‐related joint disease but also considered as long‐term mild inflammatory‐related disease mediated by inflammatory cytokines derived from abnormal genes upregulation.^[^ ^2^, ^3^, ^4^ ^]^ Generally, the mechanical and biological factors can aggravate the degenerative process collaboratively. However, current clinical treatments are restricted in nonsteroidal anti‐inflammatory drugs, lubricants, hormones, or cytotoxic drugs, which lack synergistic therapeutic effect, have toxicity with long‐term use, and most importantly, have limited efficacy to reverse the degenerative process.^[^ ^5^, ^6^ ^]^ Therefore, a synergistic therapy that simultaneously promotes intra‐articular lubrication and inhibits the up‐regulation of pro‐inflammatory genes is urgently needed for OA treatment.

The aim of gene therapy for OA is to deliver gene‐based treatments into the joints, leading to a site‐specific, controllable and long‐term presence of therapeutic agents to safeguard and rebuild the damaged articular cartilage.^[^ ^7^ ^]^ For example, CRISPR‐based gene editing, which targets IL‐1*β*, MMP‐13, or NGF, can abate aberrant genes and is proved to have excellent OA‐modifying effects.^[^ ^2^ ^]^ In addition, gapmer antisense oligonucleotides can achieve unassisted cellular entry and gene silencing to reduce COX‐2 expression, which thereby efficiently treat OA.^[^ ^8^ ^]^ Moreover, delivery of antisense oligonucleotides for ADAMTS5 inhibition is also shown to efficiently reverse OA cartilage degeneration by catabolic gene regulation.^[^ ^9^ ^]^ Previous studies have found that inhibiting the transcription and/or translation of overexpressed genes through the delivery of nucleic acids shows great promise.^[^ ^10^, ^11^ ^]^ For regulating pro‐inflammatory genes expression, IL‐1*β* is considered to be a key target because it hastens the apoptosis of chondrocytes, induces the production of matrix metalloproteinases, and accelerates the degradation of type II collagen and aggrecan.^[^ ^3^, ^12^, ^13^, ^14^ ^]^ However, traditional therapeutic oligonucleotides are unstable in microenvironment and inefficient for cell uptake. Therefore, we modified the anti‐sense DNA sequence of the mRNA of IL‐1*β* onto gold nanorods (Au NRs), as the obtained spherical nucleic acids (SNAs) are reported to have enhanced stability and transmembrane ability.^[^ ^15^, ^16^ ^]^ Based on RNA interference, post‐translational gene silencing with SNAs can achieve cytokine‐specific reduction with no need for transfection agents.

In spite of the powerful efficacy of the SNAs, the other challenges for gene therapeutics are local long‐term retention, stability, and controllable release in that a one‐time large‐dose injection of genetic drugs in joint cavity may cause safety risks. Hyaluronic acid (HA) is shown in numerous recent studies to be a viable intra‐articular treatment option and universally applied in clinic, showing great lubrication function in joint cavity and long‐term improvement in both pain and function.^[^ ^17^ ^]^ HA‐based biomaterials, such as hydrogels,^[^ ^18^, ^19^, ^20^, ^21^ ^]^ nanocarriers,^[^ ^22^, ^23^, ^24^, ^25^ ^]^ and micro‐scaffolds,^[^ ^26^, ^27^, ^28^ ^]^ are emerging as appealing starting materials due to HA's biocompatibility, native biofunctionality, biodegradability, non‐immunogenicity, and versatility.^[^ ^29^ ^]^ However, many of them degrade very fast and lack high‐dose injectability, which limits their potential biomedical applications. More importantly, the poor anti‐inflammatory property of HA limits its application in the treatment of OA.^[^ ^30^ ^]^

Here, in order to further develop a combinational therapeutic platform, we grafted HA with single‐strand DNA with the complementary sequence of SNAs. As the excellent hydrophilicity of DNA materials, we hypothesize that this DNA‐grafted HA (termed as ^DNA^HA) can improve the injectability and biostability for high‐dose injection. In addition, this ^DNA^HA can be assembled with SNAs by DNA hybridization, which endows the system with promising anti‐inflammatory effect. In the presence of the Au NRs,^[^ ^31^ ^]^ the formed ^DNA^HA‐SNAs system (termed as HA‐SNA) can be dissembled by NIR light‐induced thermo effect, thus achieving controlled SNAs release.

**Scheme 1:**
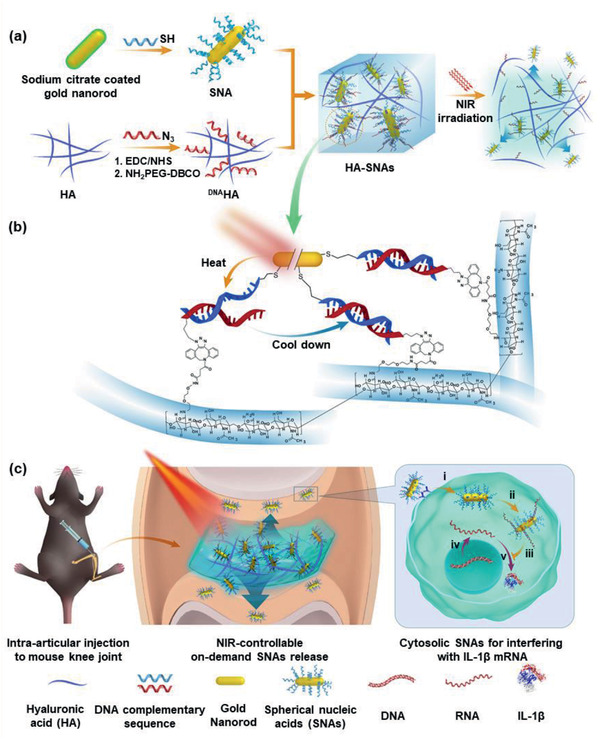
NIR light‐controllable SNAs release based on DNA‐grafted HA for OA treatment. a) Synthesis of HA‐SNAs multi‐functional delivery system via DNA hybridization and its unspooling to release SNAs by virtue of photothermal response. b) Chemical modification and base pairing design of ^DNA^HA and SNAs aimed at IL‐1*β*. c) HA‐SNAs system is injected into the knee joint and irradiated by NIR light to gradually release the SNAs, which enter into cells to interfere with mRNA molecules to silence IL‐1*β* expression. c(i)) Cell internalization; c(ii)) SNAs capturing the targeted mRNA; c(iii)) Inhibiting the process of translation; c(iv)) Transcription from DNA to mRNA; and c(v)) Translation from mRNA to IL‐1b protein.

Based on these premises, a NIR light‐triggered inflammatory gene‐targeted drug delivery system is designed and fabricated by incorporating ^DNA^HA and SNAs by DNA hybridization for local injectable gene treatment of OA, as shown in **Scheme** **1**. The features of heat sensitivity and photothermal conversion endow the HA‐SNAs with the ability of absorbing NIR light and converting it into heat to facilitate the breakdown of hydrogen bonds of DNA helix and subsequent NIR light‐responsive controllable release of the SNAs to interfere with IL‐1*β* mRNA molecules. The materials structure, NIR light‐responsive controlled release, biocompatibility, and anti‐inflammatory capability are investigated systematically to evaluate the performance of the HA‐SNAs. We hypothesize that the NIR light‐triggered thermal‐assisted gene therapy system developed here, HA‐SNAs, can be employed intra‐articularly as an effective injectable agent to inhibit the development of OA.

## 2 Results and Discussion

In order to evaluate the potential clinical application of the NIR light‐triggered thermal‐assisted gene therapy system based on HA, we performed in vitro and in vivo experiments to demonstrate whether HA‐SNAs irradiated with NIR light (termed as HA‐SNAs+NIR) protect the chondrocytes from the oxidative stress‐induced degeneration and impede the development of OA.

### 2.1 Preparation of ^DNA^HA‐SNAs System and Characterization

The cetyltrimethylammonium bromide (CTAB) Au NRs with localized surface plasmon resonance maximum wavelength at 808 nm were used as the framework for thermo‐responded SNAs preparation. The antisense DNA strands (5′‐TTGTTGTTCATCTCGGAGCTTTTT‐3′) with a C_6_SH as 3′‐terminus were then linked to the surface of Au NRs by Au‐sulfur conjugation in phosphate buffer saline (PBS) solution to form the SNAs. The loading degree of the oligonucleotide in SNAs is 1.2% ± 0.1%, detected by ultraviolet (UV) absorption at 280 nm. For ^DNA^HA preparation, firstly, the HA with the molecule weight of 970 kD was treated by DMTMM [4‐(4,6‐dimethoxy‐1,3,5‐triazin‐2‐yl)‐4‐methyl‐morpholinium chloride) for activation of carboxylic acids in MES (2‐(*N*‐morpholino) ethane sulfonic acid] buffer. Then the NH_2_‐PEG_4_‐DBCO was used as the linker to be modified onto the activated HA. Next, functionalized single‐stranded DNA (5′‐GCTCCGAGATGAATTTTT‐3′) with N_3_ at the 3′‐tuminus, which was used as the sense strand for hybridizing with the antisense DNA strands on SNAs, was conjugated onto the DBCO modified HA by strain‐promoted alkyne‐azide cycloaddition to obtain the ^DNA^HA (all the DNA strands were purchased from Sangon Biotech, China). The DNA strands are shown in Table S1, Supporting Information. Finally, the prepared SNAs and ^DNA^HA were mixed in double‐distilled water (ddH_2_O) to form the photo‐thermo responded ^DNA^HA‐SNAs system. The mass ratio of SNAs: ^DNA^HA: ddH_2_O is 2:20:100.

**Figure 1:**
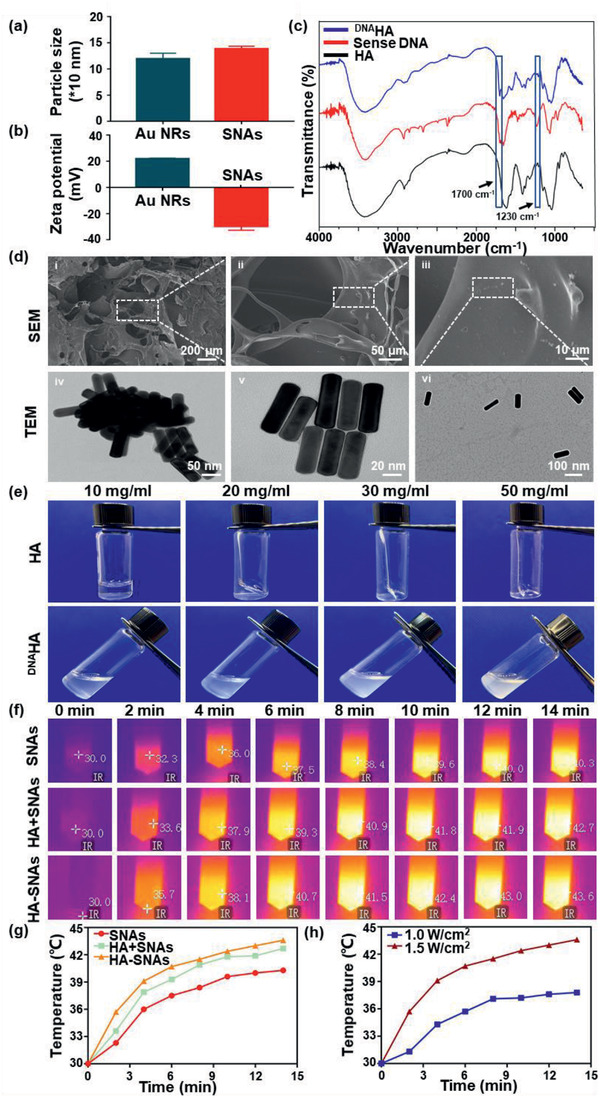
Characterization of the HA‐SNAs system. a) Size and b) zeta(*ζ*)‐potential changes of the nanosystem during the preparation. c) FTIR spectra of the HA, sense DNA, and ^DNA^HA. d) SEM images of HA‐SNAs at different magnification and the TEM images of (d(iv)) Au NPs, d(v)) SNAs, and d(vi)) HA‐SNAs. f) Photothermal images of SNAs, HA+SNAs, HA‐SNAs irradiated at 808 nm (1.5 W cm^−2^) for 14 min, respectively. g) Temperature profiles of SNAs, HA+SNAs, HA‐SNAs irradiated at 808 nm (1.5 W cm^−2^) for 14 min, respectively. h) Temperature profiles of HA‐SNAs irradiated at 808 nm for 14 min with power densities of 1.0 and 1.5 W cm^−2^, respectively.

The fabrication process is shown in Scheme 1. The size and zeta(*ζ*)‐potential changes of Au NRs during the preparation are shown in **Figure** **1**a,b. After antisense DNA conjugation, the *ζ*‐potential of CTAB coated Au NRs sharply reduced from +20 mV to −34 mV, followed by the size increase with about 15 nm. The relevant Fourier transform infrared (FTIR) data of CTAB coated Au NRs and SNAs are listed in Figure S1, Supporting Information. For ^DNA^HA, the FTIR data demonstrated successful DNA conjugation to HA, in view of the typical P–O stretching absorption band at 1230 cm^−1^ and P–O bending at 1700 cm^−1^ in the spectrum of ^DNA^HA (Figure 1c). The H^1^‐NMR detection in Figure S2, Supporting Information, further confirmed the preparation of ^DNA^HA. The atomic force microscopy (AFM) of the AuNRs and SNAs are compared in Figure S3, Supporting Information.

The relevant scanning electron microscope (SEM) and transmission electron microscopy (TEM) imaging are shown in Figure 1d. Figure 1d(i–iii) showed that Au NRs were dispersed in the system. For TEM, it can be found that pure Au NRs aggregated together (Figure 1d(iv)), while after DNA conjugation, the aggregation of the Au NRs was reduced (Figure 1d(v)). As illustrated in Figure 1d(vi), Au NRs were almost separated after mixed with ^DNA^HA, indicating that the DNA hybridization between the sense strands on HA skeleton and antisense strands on SNAs promoted the nanoparticles' separation.

In Figure 1e, HA and ^DNA^HA solution in different concentrations are compared to show that the promotion effect to injectability after DNA conjugation. Generally, the dissolution of HA in water is a time‐consuming process that gradually becomes difficult as the concentration increases, followed by an obviously drop in injectability.^[^ ^32^ ^]^ When the concentration of HA is higher than 20 mg mL^−1^, the dissolution process usually takes several hours and the final solution is gelatinous. In contrast, ^DNA^HA demonstrated rapid dissolution within several minutes. Moreover, the solution was always liquid with good fluidity, even the concentration reached 70 mg mL^−1^, indicating the DNA conjugation is an effective strategy to promote the injectability of HA for clinical application.

Then the photo‐thermo effects of the SNAs in different systems were detected (Figure 1f and Figure S4a, Supporting Information). Samples were dispersed in ultrapure water to obtain the solutions with concentrations of 20 mg mL^−1^. Then the solutions were irradiated with 808 nm laser with power densities of 1.0 and 1.5 W cm^−1^ for 14 min and the infrared thermal imaging camera was used to monitor the temperature. The temperature profiles of SNAs, HA+SNAs, HA‐SNAs irradiated at 808 nm (1.0 and 1.5 W cm^−2^) for 14 min are shown in Figure 1g and Figure S4b, Supporting Information. The temperature profiles of HA‐SNAs irradiated at 808 nm for 14 min with power densities of 1.0 and 1.5 W cm^−2^, respectively, are shown in Figure 1h. HA‐SNAs showed the fastest temperature increase, while the SNAs water solution was the slowest. This means that although the dehybridization of double‐strands DNA is an endothermic process, the changes in specific heat capacity by different polymers to water is the major reason for themo‐effect. The temperature of all samples showed 10 °C increase within 6 min under 1.0 W cm ^−2^ irradiation.

### 2.2 In Vitro Cytotoxicity

**Figure 2:**
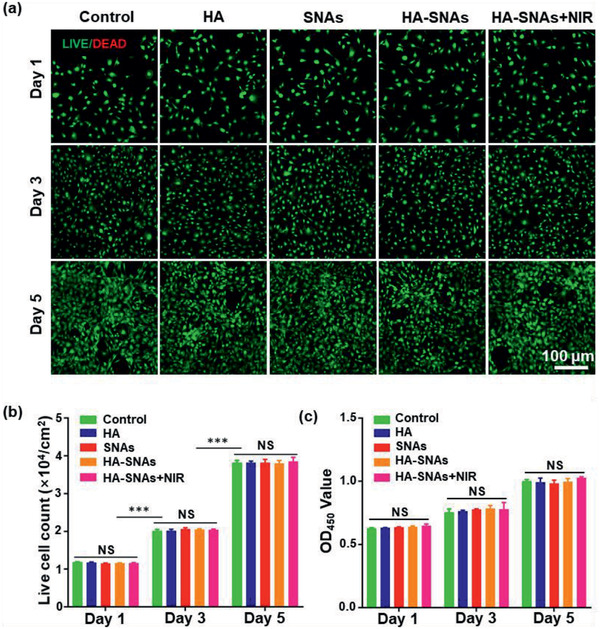
Cytotoxicity of the nanosystem in vitro. a) Live/Dead staining of chondrocytes co‐cultured with HA, SNAs, HA‐SNAs, HA‐SNAs+NIR detected employing fluorescence microscopy. b) The live cell count summarized from the Live/Dead assay. c) Cytotoxicity of HA, SNAs, HA‐SNAs, and HA‐SNAs+NIR on chondrocytes examined with CCK‐8. *n* = 3; NS = no significance; ***p < 0.001.

The in vitro cytotoxicity of HA, SNAs, HA‐SNAs, and HA‐SNAs+NIR on primary mouse chondrocytes was evaluated by Live/Dead assay and CCK‐8 test at 1, 3, and 5 days. In **Figure** **2**a, the Live/Dead staining showed that over the course of 5‐day culture, only very few dead cells were observed and most of the seeded cells stayed alive. Furthermore, from day 1 to day 5, the cell density increased gradually, which was confirmed by the incremental number of viable cells shown in Figure 2b. In addition, the CCK‐8 test showed that compared with the control group, there was no significant difference in the proliferation activity and cell viability among the experimental groups at all time points (Figure 2c). All these results attest that the HA‐SNAs has excellent biocompatibility to the chondrocytes.

### 2.3 In Vitro Photothermal Release and Cellular uptake

**Figure 3:**
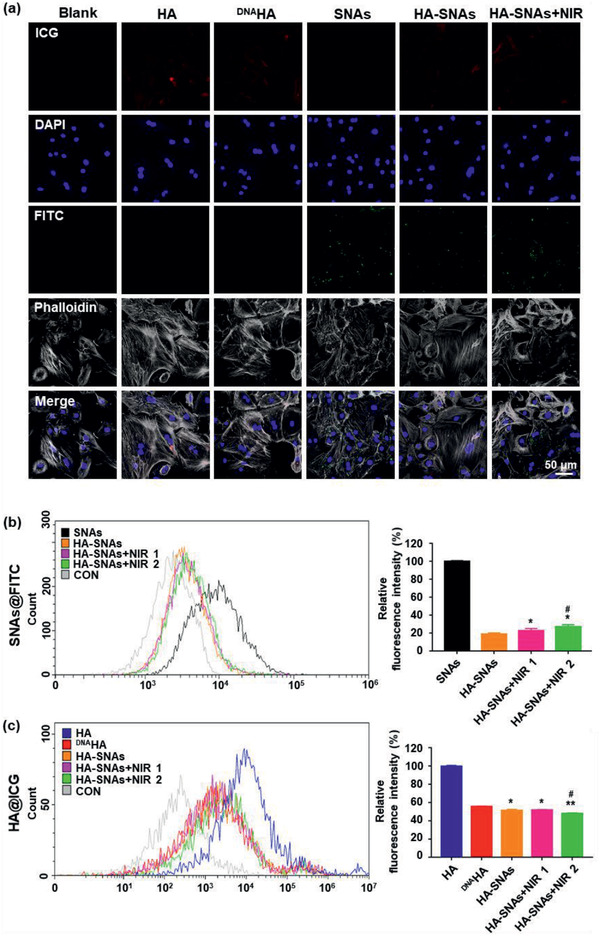
Cellular uptake. a) Representative photomicrographs of chondrocytes acquired using CLSM treated with HA, ^DNA^HA, SNAs, HA‐SNAs, HA‐SNAs+NIR for 12 h. Red: ICG labeling HA; Green: FITC labeling SNAs; blue: DAPI labeling cell nuclei; gray: Phalloidin labeling cell actin. b) Flow cytometry analysis of cellular uptake after incubating SNAs, HA‐SNAs, HA‐SNAs+NIR for 12 h with chondrocytes where SNAs are labeled with FITC with the corresponding cellular uptake quantification. *n* = 3; *p < 0.05, **p < 0.01, compared with HA‐SNAs; ^#^ *p* < 0.05, compared with HA‐SNAs+NIR 1. c) Flow cytometry analysis of cellular uptake after incubating HA, ^DNA^HA, HA‐SNAs, HA‐SNAs+NIR with chondrocytes where HA are labeled with ICG. Correspondingly, the cellular uptake is quantified. *n* = 3; *p < 0.05, **p < 0.01, compared with ^DNA^HA; ^#^ *p* < 0.05, compared with HA‐SNAs. The power density of NIR light is 1 W cm^−2^.

In order to verify the transmembrane ability of SNAs and NIR light‐responsiveness of HA‐SNAs, we co‐incubated HA, ^DNA^HA, SNAs, HA‐SNAs, and HA‐SNAs+NIR with chondrocytes to evaluate the cellular uptake and NIR‐responsive release of the nanosystem in vitro. In order to reduce the side‐effects of the photothermal approach on cells, the radiation power density of NIR light was adjusted to 1 W cm^−2^. At the same time, the NIR light was radiated via multiple cycles, which promoted the efficient release of SNAs from the HA‐SNAs (HA‐SNAs+NIR 1 for 10 min and HA‐SNAs+NIR 2 for 20 min). Confocal laser scanning microscopy (CLSM) imaging was used to visualize the process of cell uptake (**Figure** **3**a). Furthermore, flow cytometric analysis revealed that compared with pure SNAs, cell uptake signal of SNAs coupling with ^DNA^HA showed a dramatic reduction (around 80%), suggesting that the ^DNA^HA can efficiently block the interactions between cells and SNAs. After NIR irradiation, the cell uptake efficiency to SNAs demonstrated an irradiation time‐depended increase. This NIR‐triggered cell uptake behavior confirmed that the ^DNA^HA‐SNAs system was followed by a photo‐thermo induced reversible disassembly to achieve on‐demand drug release. In addition, a stronger uptake of SNAs by chondrocytes after the HA‐SNAs (18.6%) was exposed to NIR light (22.6%), where the cell uptake reached the maximum in the HA‐SNAs+NIR 2 group (27.0%), which was time‐dependent (Figure 3b).

Next, the influence of DNA grafting on cell uptake efficiency of HA was also investigated. Compared with HA group, the cell uptake to ^DNA^HA showed a great decrease (around 60%). This confirmed the hypothesis that high hydrophilic and negative‐charged properties of DNA would impede the cell uptake to ^DNA^HA,^[^ ^33^ ^]^ which was favorable for promoting the bio‐stability of the system in an in vivo application. After coupling with SNAs, the fluorescence intensity of the HA‐SNAs was further reduced; however, as the ^DNA^HA is the major part of the ^DNA^HA‐SNA system, this reduction in the fluorescence was not significant (Figure 3c). In order to further validate the transmembrane ability of SNAs, we used cell membrane specific fluorescent dye to test this (Figure S5, Supporting Information). All these results indicate a favorable transmembrane ability and NIR light‐responsiveness of the nanosystem, and also indicate that the DNA sequences modification improved the bio‐stability of HA.

### 2.4 In Vivo Joint Residence and Photothermal Responsiveness

Previous studies have found that modified HA with functional groups such as poly(ethylene glycol),^[^ ^34^ ^]^ tannic acid,^[^ ^35^ ^]^ and nanoparticles^[^ ^36^ ^]^ slowed down its degradation, promoting its injectability and endowing it with additional functions. In addition, changes in glycosidic bonds and increased steric hindrance caused by chemical modification lead to enhanced HA stability and prolonged bioavailability of the agent.^[^ ^37^ ^]^

**Figure 4:**
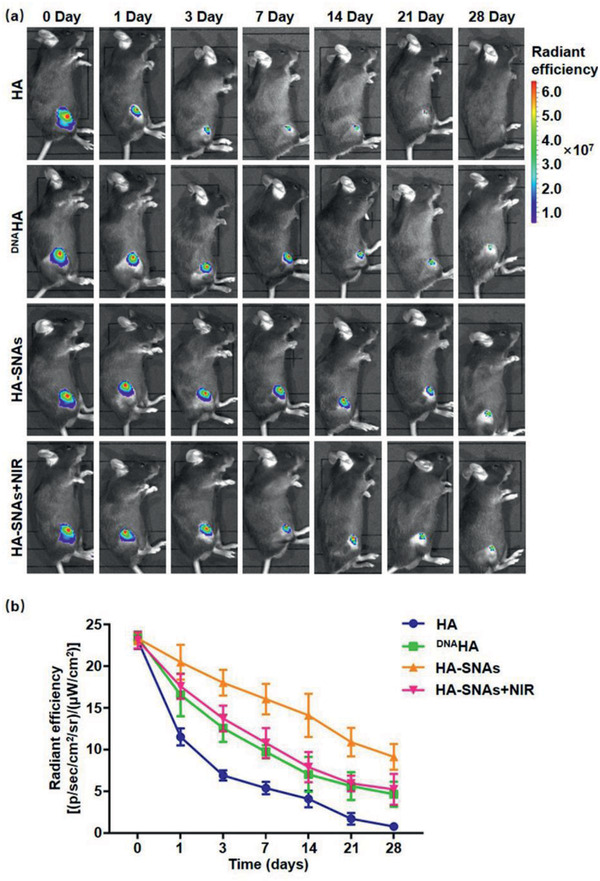
Joint residence of the injected nanosystem. (–a) Representative IVIS images of mice knee joints over 28 days after injection of fluorescent HA, ^DNA^HA, HA‐SNAs and HA‐SNAs+NIR. Fluorescence scale: max = 7.0 × 10^7^, min = 1.0 × 10^7^. b) Time course of fluorescent radiant efficiency within joints corresponding to a). *n* = 3. Half‐lives are statistically different for each dataset (*p* < 0.05).

Thus, to evaluate the photothermal release and retention time of the nanosystem in vivo we injected ICG fluorescently labeled HA, ^DNA^HA, HA‐SNAs, and HA‐SNAs+NIR intra‐articularly into the mice knees. The NIR light source of 1 W cm^−2^ lasted for 9 min per h cycling for 6 h every day. In order to ensure the quantity of fluorophore to be equal, concentrations were tuned in each formulation at first, and all formulations (about 10 mg mL^−1^ HA and about 50 µg mL^−1^ SNAs) were injected in the synovial fluid. An in vivo imaging system (IVIS) was used to serially measure the fluorescence within joints over a period of 1 month (**Figure** **4**a). The total radiant efficiency data within the anatomical region of interest (ROI) were quantified and plotted over time (Figure 4b). The individual HA showed a significant drop after 24 h, and further dropped by 76.7% after 1 week, whereas in comparison, the decline trend of ^DNA^HA significantly slowed down, and 30.0% still left in the joint after 2 weeks. Moreover, after coupling with SNAs, the system is more stable, with 39.1% remaining after 28 days. This proved that DNA grafting and SNAs coupling to HA can considerably promote the biostability of HA and extend the relevant retention time in knee joints. Furthermore, it can be observed that the downward trend of the HA‐SNAs signal speeded up following repeating NIR light radiation, indicating that the release of SNAs would accelerate the degradation of the entire system in the joints. All these results demonstrate that DNA sequences and SNAs modification considerably extended joint residence time of the HA, while NIR light‐responsive release of SNAs accelerated degradation of the nanosystem.

### 2.5 In Vitro Protective Effect of Chondrocytes Degeneration

**Figure 5:**
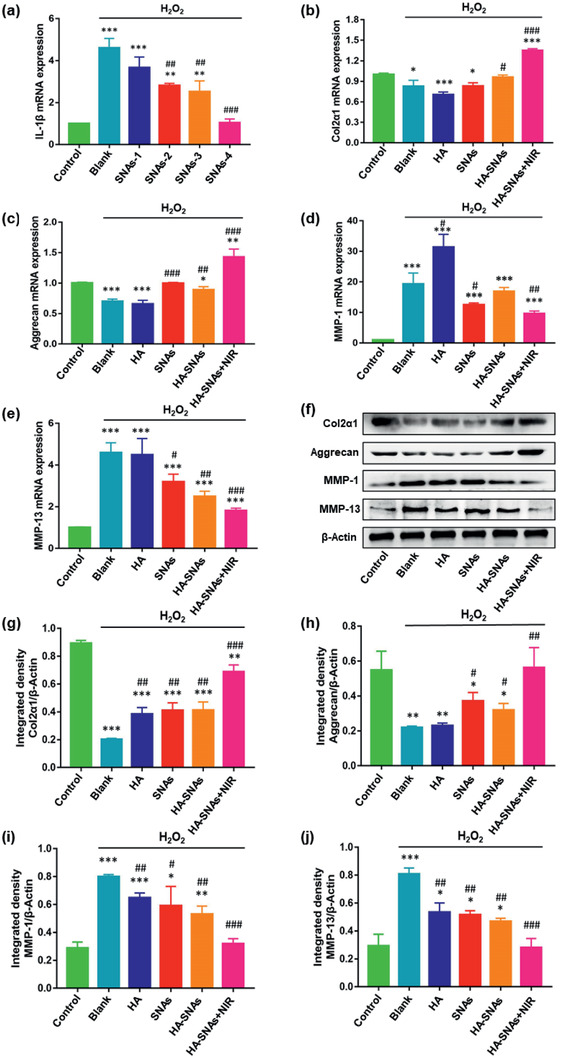
Mechanism for chondrocytes degradation protection of the nanosystem. a) The qRT‐PCR analysis exhibiting the mRNA expression of IL‐1*β* in chondrocytes treated with 10 mU of H_2_O_2_ at different concentrations of SNAs (0, 5, 10, 20, and 50 µg mL^−1^). *n* = 3, **p < 0.01, ***p < 0.001, compared with control; ^##^ *p* < 0.01, ^###^ *p* < 0.001, compared with blank group. qRT‐PCR analysis and quantitation of b) Col2*α*, c) aggrecan, d) MMP‐1, and e) MMP‐13 expression in chondrocytes treated with 10 mU of H_2_O_2_, and cocultured with HA, SNAs, HA‐SNAs, HA‐SNAs+NIR for 24 h. f) Western blot analysis and quantitation of g) Col2*α*, h) aggrecan, i) MMP‐1, and j) MMP‐13 expression in chondrocytes treated with 10 mU of H_2_O_2_, and cocultured with HA, SNAs, HA‐SNAs, HA‐SNAs+NIR for 24 h. *n* = 3, *p < 0.05, **p < 0.01, ***p < 0.001, compared with control; ^#^ *p* < 0.05, ^##^ *p* < 0.01, ^###^ *p* < 0.001, compared with blank group.

The pathogenesis of OA is associated with multiple factors, such as inflammatory factors, mechanical loading stress and reactive oxygen species (ROS). These factors can lead to the degeneration of chondrocytes, which is regarded as the most significant characteristic of OA. Furthermore, the degradation of cartilage matrix components (mainly Col2*α* and aggrecan) also plays an important role in OA. It results in the loss of cartilage function and structure, where IL‐1*β*‐induced MMP‐13 and MMP‐1 expression work.^[^ ^38^ ^]^ In this study, 10 mU of H_2_O_2_ was introduced as reported by Yan et al,^[^ ^39^ ^]^ to simulate the ROS stress of chondrocytes, which was essential for the pathogenesis of OA. The mRNA expression of IL‐1*β* increased significantly in chondrocytes treated with 10 mU of H_2_O_2_ and decreased gradually after addition of SNAs at increasing concentrations of 0, 5, 10, 20, and 50 µg mL^−1^. Respectively, the IL‐1*β* gene expression silencing efficiency were 79%, 61%, 55% and 23% (**Figure** **5**a). In order to explore the protective effect of HA‐SNAs+NIR for chondrocytes degeneration induced by H_2_O_2_, subsequently we evaluated the expression levels of Col2*α*, aggrecan, MMP‐1 and MMP‐13 in chondrocytes after addition of HA, SNAs, HA‐SNAs, and HA‐SNAs+NIR. And chondrocytes in the blank group were merely treated with H_2_O_2_. The quantitative real‐time polymerase chain reaction (qRT‐PCR) analyses indicated that the addition of HA‐SNAs+NIR reversed the decrease in Col2*α* and aggrecan mRNA expression levels in comparison with the blank group. In contrast, compared with the blank group, the mRNA expression levels of MMP‐1 and MMP‐13 greatly decreased after the addition of HA‐SNAs+NIR (Figure 5b–e). Western blot analysis indicated that the expression levels of Col2*α* and aggrecan increased significantly while MMP‐1 and MMP‐13 decreased significantly after addition of HA‐SNAs+NIR. All these results indicated that the HA‐SNAs+NIR can protect chondrocytes from degeneration (Figure 5f–j).

**Figure 6:**
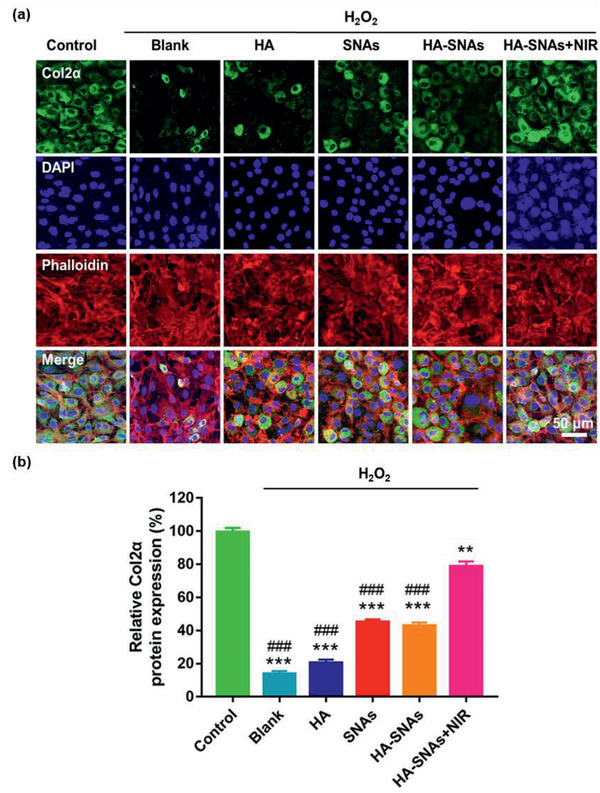
Immunofluorescence staining. a) Representative photomicrographs of chondrocytes treated with 10 mU of H_2_O_2_ and co‐cultured with HA, SNAs, HA‐SNAs, HA‐SNAs+NIR for 12 h, acquired using a CLSM. Green: Molecular Probes labeling Col2*α*; red: Phalloidin labeling cell actin; blue: DAPI labeling cell nuclei. b) The quantitative data showing comparison of Col2*α* protein expression of chondrocytes treated with 10 mU of H_2_O_2_, and co‐cultured with HA, SNAs, HA‐SNAs, HA‐SNAs+NIR for 12 h. *n* = 3, **p < 0.01, ***p < 0.001, compared with control; ###*p* < 0.001, compared with HA‐SNAs+NIR.

Furthermore, immunofluorescence staining was used to examine the protein expression level of Col2*α* as a representative in chondrocytes, which were treated with H_2_O_2_ and co‐cultured with HA, SNAs, HA‐SNAs, and HA‐SNAs+NIR. As demonstrated in **Figure** **6**, the staining intensity of the control group was visibly higher than that of the blank group. That meant the protein expression level of Col2*α* significantly decreased following treatment with H_2_O_2_ (Figure 6a). Compared with the blank group, the protein expression level of Col2*α* significantly increased by 4.6‐fold after addition of HA‐SNAs+NIR (Figure 6b). All these results show that the NIR light‐triggered thermal‐assisted gene therapy system developed here can protect the chondrocytes from oxidative stress‐induced degradation, which was achieved by down‐regulating the catabolic proteases (MMP‐1, MMP‐13) and up‐regulating the anabolic components (Col2*α*, aggrecan) of articular cartilage.

### 2.6 In Vivo Therapeutic Effect of OA

Destabilization of the medial meniscus (DMM)‐induced OA has been commonly employed as an animal model.^[^ ^40^ ^]^ Here, X‐ray radiography, micro‐computed tomography (micro‐CT) scanning and reconstruction, histological and immunohistochemistry staining were performed to analyze C57 mice that had undergone DMM surgery and received different treatments. First, fourth, seventh and tenth week, respectively, after the DMM surgery, PBS, HA, SNAs, HA‐SNAs, and HA‐SNAs+NIR were injected once into the mice knee joints. In the HA‐SNAs+NIR group, an 808 nm NIR light (1 W cm^−2^) was used to irradiate the knee joints for 9 min per h cycling for 6 h a day. Before irradiating NIR light, all mice were forced to run on the treadmill for 1 h to simulate an acute phase of OA. The NIR light was administered once every week until twelve weeks. The animal handling is shown in Figure S6, Supporting Information.

**Figure 7:**
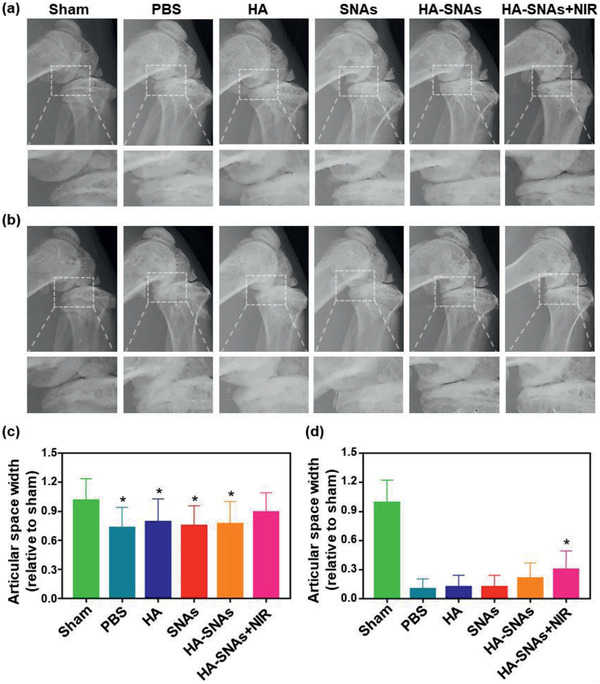
X‐ray radiography. Representative X‐ray radiographs of the mice knee joints showing the treatment of DMM‐induced OA after the intra‐articular injection of PBS, HA, SNAs, HA‐SNAs, HA‐SNAs+NIR at a) 4 weeks and at b) 12 weeks after surgery. The relative articular space width between the medial compartments of mouse knee joints at c) 4 weeks and at d) 12 weeks after surgery. *n* = 5, *p < 0.05, compared with the PBS group.

At fourth and twelfth weeks following the DMM surgery, the X‐ray radiographs were obtained and the articular space width of the mice knee joints is shown in **Figure** **7**. As shown in Figure 7a,b, the typical OA feature, such as joint space narrowing, was intuitively observed in the PBS group. From the radiographs, the values of articular space width can be calculated, and it was observed that the medial compartments of the mice knee joints became narrower at 12 weeks, following DMM surgery for all the groups. However, a slightly larger articular space width was observed in the HA‐SNAs+NIR group (Figure 7c,d).

**Figure 8:**
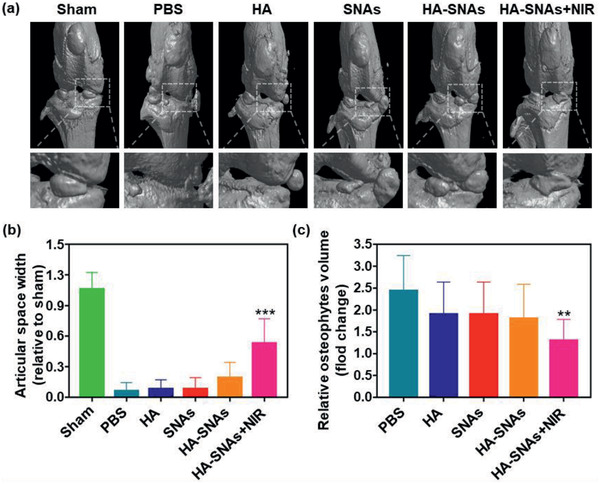
Micro‐CT arthrography. a) Representative micro‐CT scanning and reconstruction of mouse knee joints showing the treatment of DMM‐induced OA after the intra‐articular injection of PBS, HA, SNAs, HA‐SNAs, HA‐SNAs+NIR at 12 weeks after surgery. b) The relative articular space width between the medial compartments of mice knee joints at 12 weeks after surgery. c) The relative osteophytes volume of the experimental groups. *n* = 5, **p < 0.01, ***p < 0.001, compared with the PBS group.

Subsequently, based on micro‐CT scanning and reconstruction, the mice knee joints were carefully dissected and further examined, as shown in **Figure** **8**. In comparison with the X‐ray radiographs, the micro‐CT imaging of joint structures demonstrated a more detailed and conspicuous characteristics of OA. As shown in Figure 8a, extensive pathological changes, such as joint space narrowing, articular cartilage destruction, subchondral bone sclerosis and osteophyte formation, were observed in the PBS group. In terms of quantitative analysis, the value of articular space width and the number of osteophytes were both calculated. And as similar as the X‐ray, it was indicated that the medial compartments of the mice knee joints became narrower for all the groups. However, a significantly larger articular space width was observed in the HA‐SNAs+NIR group (Figure 8b). In addition, although the osteophyte volume increased for all the groups, the value of the HA‐SNAs+NIR group was slightly lower in comparison to other groups (Figure 8c). The above results indicate that the synergistic therapy, which simultaneously promotes intra‐articular lubrication and inhibits the up‐regulation of pro‐inflammatory genes, efficiently inhibits the development of OA.

**Figure 9:**
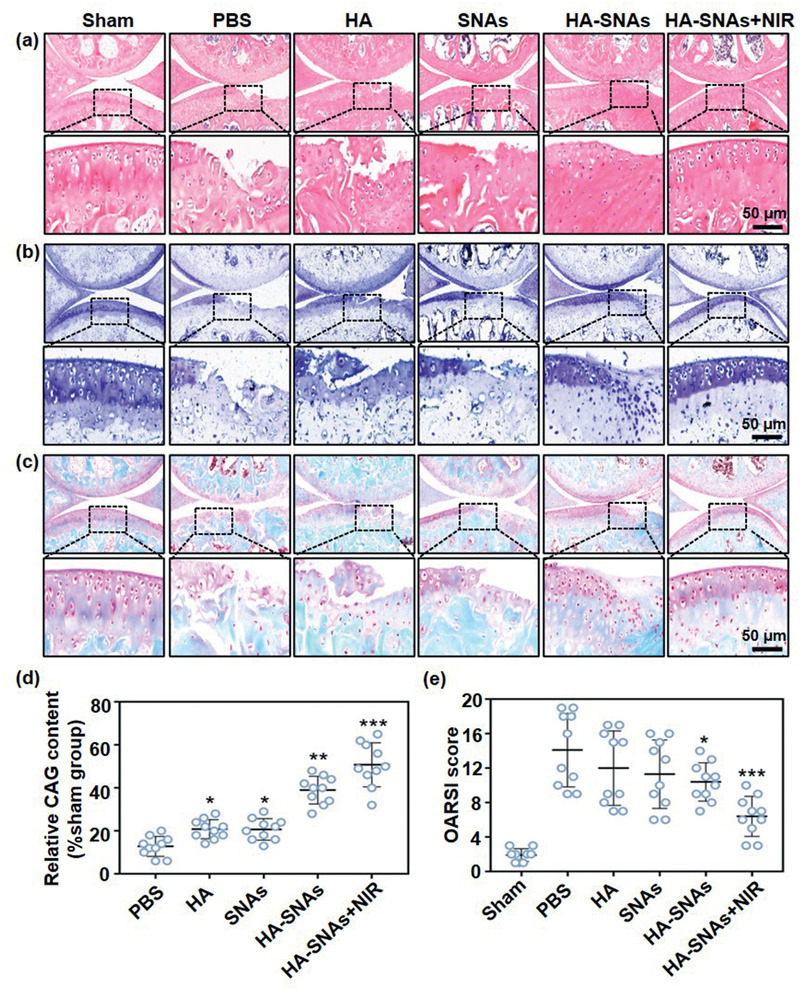
Histological staining. a) Representative H&E staining, b) Toluidine Blue staining, and c) Safranin O‐fast green staining of the cartilage sections showing the treatment of mouse DMM‐induced OA after injection of PBS, HA, SNAs, HA‐SNAs, and HA‐SNAs+NIR at 12 weeks after surgery. d) GAG content relative to the sham group obtained from the quantitative analysis of Safranin O‐fast green staining of the cartilage sections using the Image J software. e) OARSI score of articular cartilage for each group after treatment for 12 weeks. *n* = 10. *p < 0.05, **p < 0.01, ***p < 0.001, compared with the PBS group.

**Figure 10:**
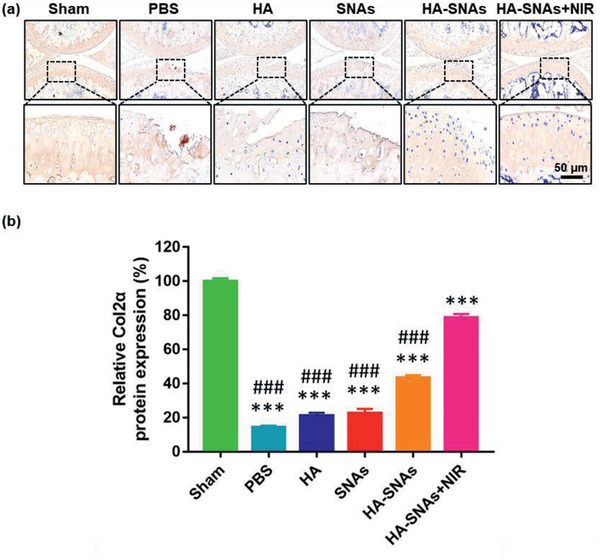
Immunohistochemistry staining. a) Representative fluorescence images showing the protein expression level of Col2*α* in the articular cartilage of mice knee joints after intra‐articular injection of PBS, HA, SNAs, HA‐SNAs and HA‐SNAs+NIR at 12 weeks following the DMM surgery. b) The quantitative data showing the protein expression level of Col2*α* acquired from the fluorescence intensity using the Image J software. *n* = 10, the values are presented as mean ± SD, ***p < 0.001, compared with the Sham group; ^###^ *p* < 0.001, compared with the HA‐SNAs+NIR group.

Hematoxylin‐eosin (H&E) staining, Toluidine Blue staining and Safranin O‐fast green staining were then performed to evaluate histologically the cartilage tissues. As shown in **Figure** **9**a–c, typical OA features, such as vertical fissure, erosion denudation, surface discontinuity and deformation, were observed in the PBS group. With respect to matrix staining, morphological change and tidemark integrity promotion, all the treatment groups presented varying degrees of improvement in comparison to the PBS group. Specifically, the HA‐SNAs+NIR group was considered as the most powerful in maintaining the columnar architecture of normal cartilage, which was typically manifested as decreased surface denudation and deformation, increased tissue cellularity and cloning, as well as less severe lesion and extensive erosion. In addition, more intense Safranin O‐fast green positive staining was observed in the HA‐SNAs+NIR group in comparison to the other groups (Figure 9d). This finding indicated that the HA‐SNAs+NIR group performed better in terms of attenuation of cartilage matrix depletion, retention of overall cartilage thickness, as well as glycosaminoglycan (GAG) deposition. The result of OA Research Society International (OARSI) score is showed in Figure 9e. The OARSI scores decreased more or less in all the treatment groups in comparison to the PBS group, but the HA‐SNAs+NIR group presented the best outcome with about 55% reduction in the OARSI score. Furthermore, the immunohistochemistry staining was performed to detect Col2*α* protein expression level in chondrocytes of the cartilage tissues (**Figure** **10**). At twelfth week following the DMM surgery, the Col2*α* protein expression level significantly decreased as the staining intensity of the Sham group was visibly higher than that of the PBS group (Figure 10a). The addition of HA‐SNAs+NIR significantly increased the Col2*α* protein expression level in comparison to that of other groups (Figure 10b). All these results indicate that the HA‐SNAs+NIR group, which combines lubrication of the HA and inflammatory gene silencing of the SNAs, along with long‐term non‐invasive intervention via NIR light‐responsive release, can inhibit the development of OA.

## 3 Conclusion

Overall, we successfully designed and developed a ^DNA^HA‐SNAs system, which can perform a NIR light‐responsive on‐demand release and reversible assembly behavior followed by photo‐thermal induced DNA hybridization/dehybridization. The DNA grafting to HA promotes the injectability and bio‐stability of HA, by which overcomes the major limitations for the clinical application of HA as drug delivery system. Furthermore, after coupling with controlled release units, such as AuNRs, the endowed stability and controllability set the stage for injectable local gene delivery with safety, which is a primary concern and challenge for current local gene therapy. The in vitro and in vivo experiments showed that the NIR light‐triggered thermal‐assisted gene therapy with anti‐inflammatory ability, high‐dose injectability and long‐term stability not only protected the chondrocytes from oxidative stress‐induced degeneration, but also inhibited the development of OA based on a mouse DMM model. The mechanism was attributed to synergistic effect of long‐term lubrication and anti‐inflammation, resulting in down‐regulation of catabolic proteases and up‐regulation of anabolic components of the articular cartilage. We anticipated that such a photothermal gene and physical therapy would create a novel designing concept and application for OA localized and minimally invasive treatment.

## 4 Experimental Section

##### Synthesis of ^DNA^HA

100 mg sodium hyaluronate was dissolved in 50 mL of 0.001M HA for 1 h, and then the solution was dialyzed for 48 h. After lyophilized, the product was dissolved in 50 mL anhydrous DMSO, then 5 mg 1‐ethyl‐3‐(3‐(dimethylamino)propyl)‐carbodiimide (EDC) and 8 mg *N*‐hydroxysuccinimide (NHS) was added, the mixture was stirring for 6 h. Then 5.6 mg NH_2_‐PEG4‐DBCO was added to the solution, the reaction then lasted for 24 h with stirring. The product was then dialyzed for 72 h in deionized water. After lyophilized, the product was dissolved in 50 mL diethylpyrocarbonate (DEPC) treated water, then 10 mg sense DNA was added. The reaction was last 48 h. Then the product dialyzed for 48 h in DEPC water and then lyophilized. The obtained ^DNA^HA was stored under −20 °C.

##### Synthesis of the AuNRs‐Based SNAs

10 mg CTAB coated AuNRs was separated in 10 mL Tris (2‐carboxyethyl) phosphine hydrochloride (TCEP) treated Tris buffer (pH = 7.2). Then 10 mg sense DNA was added. The reaction lasted 24 h, and then the product was harvested by centrifugation at 13000 g for 5 min.

##### Preparing the HA‐SNAs

10 mg SNAs were separated in 10 mL of ddH_2_O, and then combined with 100 mg ^DNA^HA. The solution was heated to 80 °C for 1 h and then cooled down to 25 °C, then stored under −20 °C.

##### Characterization of the System

Zetasizer Nano ZS (Malvern Instruments Ltd.) was used to measure the hydrodynamic size (z‐average) and zeta(*ζ*)‐potential distribution of the Au NRs. Then, freeze‐dried HA‐SNAs were adhered on an aluminum substrate using carbon tape and sprayed a small amount of gold for 30 s. The coated samples were observed by SEM (HITACHI S‐4800). Under an acceleration voltage of 120 kV, TEM was used to characterize the structure of the prepared materials. The samples were prepared by depositing them onto carbon‐coated copper grids (300 mesh; Electron Microscopy Sciences, USA) and contrasting with 2% uranyl acetate solution. Before the TEM imaging, the samples coated grids were dried at room temperature.

##### Photothermal Effect and Photothermal Conversion Efficiency

Samples were dispersed in ultrapure water to obtain the solutions with concentrations of 20 mg mL^−2^. Then the solutions were illuminated with 808 nm laser with power densities of 1.0 and 1.5 W cm^−2^ for 14 min and the FLIR infrared thermal imaging camera was used to monitor the temperature changes. The temperature of HA‐SNA system showed 10 °C increasement within 6 min under 1.0 W cm^−2^ irradiation. In the in vitro experiments, cells in the HA‐SNAs+NIR group were irradiated with an 808 nm laser (1 W cm^−2^) for 9 min per h cycling for 6 h per day. In the in vivo experiments, to avoid the heat damage to normal tissues, the knee joints in mice were irradiated by an 808 nm NIR light source (1 W cm^−2^) for 9 min per h cycling for 6 h per day. NIR illumination with 1 W cm^−2^ for 9 min was used to increase the temperature to about 42 °C in the knee joints of mice treated with HA‐SNAs.

##### Primary Mouse Chondrocytes Isolation

As previously reported,^[^ ^41^ ^]^ chondrocytes were isolated from the articular cartilage of mice knee joints. The articular cartilage tissues were cut into small pieces (1 mm^3^), digested with 0.25% trypsin for 30 min and digested with 0.2% type II collagenase for 4 h. The DMEM/F12 media supplemented with 10% fetal bovine serum and antibiotics was then used to culture the obtained chondrocytes. In order to preserve the chondrocytes’ phenotype, only the chondrocytes with less than three passages were used in this study. Unless stated otherwise, the HA‐SNAs and HA‐SNAs+NIR used in the following experiments were prepared with the HA concentration of 10 mg mL^−1^, the SNAs concentration of 50 µg mL^−1^ and the NIR light source of 1 W cm^−2^ for 9 min per h cycling for 6 h per day.

##### Cell Cytotoxicity

HA, SNAs, HA‐SNAs, HA‐SNAs+NIR were co‐cultured by chondrocytes, which were seeded into a 96‐well plate at a density of 10^4^ cells per mL. The incubator at 37 °C and 5% CO_2_ was used to incubate the plate. Each well of the plate was added with 10 µL of CCK‐8 solution (ck04, Dojindo, Japan) and then the cells were cultured for another 2 h, following culturing for 1, 3, and 5 days. Then, a microplate reader (Infinite F50, Tecan, Switzerland) at a wavelength of 450 nm was used to measure the absorbance of the solution.

##### Live/Dead Staining Assay

Chondrocytes were seeded and cultured the same as before. Then, a Live/Dead Cell kit (Life Tech, USA) was used to analyze the cell viability of the HA‐SNAs group. Based on a laser scanning confocal microscope (LSCM, Zeiss, Germany), the cells were stained with 500 µL of Live/Dead cell dye for 15 min and observed, following being cocultured with HA, SNAs, HA‐SNAs, HA‐SNAs+NIR in triplicate for 1, 3, and 5 days. The viable cells with esterase activity appeared green, while the dead cells with compromised plasma membranes appeared red, as described in the manufacturer's protocol.

##### Flow Cytometry

Briefly, 10^6^ chondrocytes were incubated with FITC labeled SNAs (including SNAs, HA‐SNAs, HA‐SNAs+NIR 1 and HA‐SNAs+NIR 2) in 200 µL of DMEM/F12 for 1 h at 37 °C, respectively. And 10^6^ chondrocytes were incubated with ICG labeled HA (including HA, ^DNA^HA, HA‐SNAs, HA‐SNAs+NIR 1 and HA‐SNAs+NIR 2) in 200 µL of DMEM/F12 for 1 h at 37 °C. Chondrocytes were co‐cultured with nothing as the control group. The cells were all analyzed by flow cytometry (FACS AriaIII/BD cytometer, America), following washing three times with PBS. The irradiated two groups were irradiated with an 808 nm laser (1 W cm^−2^) for 30 s every 2.5 min (the HA‐SNAs+NIR 1 group for 10 min, and the HA‐SNAs+NIR 2 group for 20 min).

##### Intra‐Articular Injection and Metabolism

Eight‐week‐old male C57 mice were anesthetized under the isoflurane, scraped off on both legs, and disinfected with three scrubbing routines of povidone iodine and 70% (v/v) ethanol. HA, ^DNA^HA, HA‐SNAs and HA‐SNAs+NIR were injected into the right leg of the mice intra‐articularly. The fluorescence within each joint was serially acquired and quantified by an IVIS (Spectrum, PerkinElmer) and Living Image software (Caliper) over a period of 28 days. In order to ensure an equal quantity of fluorophore, concentrations were tuned in each formulation at first. Radiant efficiency data [units shown in Equation (1)] within a fixed anatomical ROI were recorded over time. The distribution of fluorescently labeled formulations was observed in mice knees 0, 1, 3, 7, 14, 21, and 28 days after injection.

(1) $$\frac{p\cdot s^{-1}\cdot cm^{-2}\cdot sr^{-1}}{\mu W\cdot cm^{-2}}$$

##### Quantitative Real‐Time PCR Analysis

At a density of 5 × 10^5^ cells per mL, chondrocytes were seeded into 6‐well plates and treated with 10 mU of H_2_O_2_ at deferent concentrations of SNAs (0, 5 µg mL^−1^, 10 µg mL^−1^, 20 µg/mL^−1^, and 50 µg mL^−1^), and co‐cultured with HA, SNAs, HA‐SNAs, HA‐SNAs+NIR for 24 h. TRIzol reagent (Invitrogen, USA) was used to extract the total RNA from the cells according to the manufacturer's protocol. The absorbance at 260 and 280 nm was measured respectively to determine the RNA concentration and purity. A Reverse Transcription System Kit (TaKaRa, China) and 1 µg of RNA were used to synthesize complementary DNA (cDNA). An ABI 7500 Sequencing Detection System (Applied Biosystems, USA) and a SYBR Premix Ex Tag Kit (TaKaRa, China) were used to perform qRT‐PCR was to amplify cDNA. By using specific primers, the expression levels of mRNA including IL‐1*β*, Col2*α*, aggrecan, MMP‐1, MMP‐13 and GAPDH were determined and normalized to GAPDH.

GAPDH: forward, 5′‐ GTGCTATGTTGCTCTAGACTTCG ‐3′; reverse, 5′‐ ATGCCACAGGATTCCATACC‐3′; Col2*α*: forward, 5′‐ TACTGGAGTGACTGGTCCTAAG‐3′; reverse, 5′‐AACACCTTTGGGACCATCTTTT‐3′; aggrecan: forward, 5′‐ TATGATGTCTACTGCTACGTGG‐3′; reverse, 5′‐GTAGAGGTAGACAGTTCTCACG‐3′; MMP‐1: forward, 5′‐GGAGGAAGGCGATATTGTGCTCTC‐3′; reverse, 5′‐CTGCTGTTGGTCCACGTCTCATC‐3′; MMP‐13: forward, 5′‐CTTCCTGATGATGACGTTCAAG‐3′; reverse, 5′‐GTCACACTTCTCTGGTGTTTTG‐3′. IL‐1*β*: forward, 5′‐ CAACTGTTCCTGAACTCAACTG‐3′; reverse, 5′‐GAAGGAAAAGAAGGTGCTCATG‐3′.

##### Western Blotting Analysis

The expression of Col2*α*, aggrecan, MMP‐1, MMP‐13 was examined by Western blotting analysis. Briefly, 10% sodium dodecyl sulfate polyacrylamide gel electrophoresis (SDS‐PAGE) was used to extract 15 µg of plasma proteins from chondrocytes. Proteins were then electroblotted onto a polyvinylidene difluoride membrane (0.45 µm; Millipore, Bedford, MA, USA). Then, they were blocked by using 5% non‐fat dry milk in Tris‐buffered saline with Tween 20 for 1 h. Subsequently, the membranes were incubated at 4 °C overnight with anti‐COL2*α*1 antibodies (1:5000 dilution), anti‐Aggrecan antibodies (1:100 dilution), anti‐MMP‐1 antibodies (1:1000 dilution) and anti‐MMP‐13 antibodies (1:3000 dilution) (all from Abcam), followed by incubation with HRP‐conjugated secondary antibodies (1:4000 dilution) (Santa Cruz) at room temperature for 1 h. The antigen–antibody complexes were then visualized by enhanced chemiluminescence assay (Thermo Scientific, Pierce, Rockford, IL, USA).

##### Immunofluorescence Staining

At a density of 5 × 10^4^ cells per well, chondrocytes were seeded onto sterile glass coverslips in 24‐well plates with addition of 10 mU of H_2_O_2_, and co‐cultured with HA, SNAs, HA‐SNAs, HA‐SNAs+NIR for 12 h. The cells were washed with PBS following incubation and then fixed in 4% paraformaldehyde for 10 min. 0.1% Triton X‐100 was then used to treat cells for 15 min, and the cells were incubated at room temperature in 3% bovine serum albumin/PBS for 30 min. Subsequently, rat primary anti‐Col2*α* antibody (Abcam, 1:200 dilution) was used to incubate with the cells at 4 °C overnight after they were washed with PBS. After incubation, appropriate Alexa Fluor 488 conjugated secondary antibodies (Molecular Probes, Life Tech, USA, 1:400 dilution) were used to incubate with the cells at room temperature for 1 h after they were washed with PBS again. The actin rings and cellular nuclei were counterstained by the Alexa Fluor 594 phalloidin (Life Tech, USA) and 4, 6‐diamidino‐2‐phenyindole dilactate (DAPI, Life Tech, USA), respectively. The CLSM was used to acquire the immunofluorescence images. Based on the fluorescence intensity of the images, the Image J software was used for quantitative comparison of protein expression level of Col2*α*.

##### Mouse OA Model and Surgical Treatment

The animal study conformed to the National Institutes of Health Guidelines for the Care and Use of Laboratory Animals and it was approved by the Animal Research Committee of Ruijin Hospital, School of Medicine, Shanghai Jiaotong University, China. The DMM surgery was used to create an OA model using C57 mice (8 weeks old, *n* = 30), as previously reported.^[^ ^40^ ^]^ Briefly, by intraperitoneal injection (30 mg kg^−1^ body weight), the pentobarbital sodium was used to anesthetize mice, and then a skin incision was made from the distal patella to the proximal tibial plateau in the right knee joint. The joint capsule medial to the patellar tendon was immediately incised with a shovel and spread open with scissors. Afterward, the cranial meniscotibial ligament of the medial meniscus (MMTL) was exposed after the fat pad was bluntly dissected and the patellar tendon was retracted. A micro‐surgical knife was used to section the MMTL, and then the wound was stitched cutaneously and disinfected with povidone iodine. The free access to food and water was provided to the mice, and the unrestricted activity was also allowed for them every day. The mice were randomly sorted into six groups (*n* = 5 for each group), and PBS, HA, SNAs, HA‐SNAs, HA‐SNAs+NIR were intra‐articularly injected into the mice knee joints once respectively at the 1st, 4th, 7th, and 10th week. In the HA‐SNAs+NIR group, an 808 nm NIR light source (1 W cm^−2^) was used to irradiate the knee joints for 9 min per h cycling for 6 h per day. Before irradiating NIR light, all mice were forced to run at a speed of 20 m min^−1^ on a level treadmill for 1 h to simulate an acute phase of OA. The NIR light was administered once every week until twelve weeks.

##### X‐Ray Radiography and Micro‐CT Arthrography

An X‐ray imager for small animals (Faxitron X‐ray, USA) was employed to perform X‐ray scanning for mice at 4 and 12 weeks, following the DMM surgery. An exposure time of 10 s was performed for the scanning with a voltage of 32 kV. The articular space width of mice knee joints was measured based on the X‐ray radiographs. Furthermore, the mice were sacrificed and the knee joint samples were obtained at 12 weeks, following the DMM surgery. A high‐resolution micro‐CT imaging system (SkyScan 1172, Bruker BioSpin, Belgium) was used to perform an arthrography to the samples. Based on the result of micro‐CT scanning and reconstruction, the articular space width and the relative osteophytes volume were evaluated.

##### Histological Staining and Immunohistochemistry Staining

After the mice were sacrificed, the knee joint samples were fixed in 4% paraformaldehyde. Then, they were decalcified in 10% ethylenediaminetetraacetic acid (EDTA) for 2 months, followed by dehydration in ethanol and embedding in paraffin. Subsequently, the paraffins loaded with samples were sectioned to a thickness of 5 µm, which were prepared for histological staining. The histopathological features were evaluated via H&E staining, Toluidine Blue staining and Safranin O‐fast green staining, and the corresponding scores were made, according to the OARSI criterion established by Pritzker et al.^[^ ^42^ ^]^ Two of the co‐authors (Chen and Qi) graded the sections independently after the digital images were captured. In addition, based on the safranin O‐fast green staining, the relative GAG content was measured using Image J software. Subsequently, in order to perform immunohistochemistry staining, the paraffin sections were deparaffinized in xylene, hydrated in ethanol, and rinsed in distilled water. Then, the sections were rinsed for three times with PBST (PBS with Tween‐20) and blocked with normal serum. As the same procedure mentioned above, the sections were incubated with mice primary anti‐Col2*α* antibodies. Subsequently, the sections were added with biotinylated secondary antibodies, and then a peroxidase‐labeled streptavidin–biotin staining technique (DAB Kit, Invitrogen, Paisley, UK) was performed. Based on the fluorescence intensity of the images, the Image J software was used to analyze protein expression level of Col2*α*.

##### Statistical Analysis

Similar independent experiments were repeated at least three times with three replicates to verify the results, and the data were shown as mean ± standard deviation (SD). The multiple comparison tests were analyzed by one‐way analysis of variance (ANOVA). The significant differences between two groups were compared by a two‐tailed nonpaired Student's *t*‐test, and the statistical significance was displayed as *p < 0.05, ** *p* < 0.01, or *** *p* < 0.001; #*p* < 0.05, ^##^ *p* < 0.01, or ^###^ *p* < 0.001.

## Conflict of Interest

The authors declare no conflict of interest.

## Supporting information

Supporting Information

## Data Availability

Research data are not shared.
