# Supplementary material for: DNA‐Grafted Hyaluronic Acid System with Enhanced Injectability and Biostability for Photo‐Controlled Osteoarthritis Gene Therapy
Source: Adv Sci (Weinh). 2021 Mar 1;8(9):2004793. doi: 10.1002/advs.202004793 (PMC8097319; doi:10.1002/advs.202004793)
Supplement: Supplementary file 1 — Supporting Information [file ADVS-8-2004793-s001.pdf]

© 2021 Wiley-VCH GmbH

ADVANCED SCIENCE
Open Access

Supporting Information

for *Adv. Sci.,* DOI: 10.1002/advs.202004793

DNA-Grafted Hyaluronic Acid System with Enhanced Injectability and Biostability for Photo-Controlled Osteoarthritis Gene Therapy

Zhijie Chen, Feng Zhang, Hongbo Zhang, Liang Cheng, Kaizhe Chen, Jieliang Shen, Jin Qi, Lianfu Deng, Chuan He*, Hélder A. Santos*, Wenguo Cui*

Supporting Information

DNA-Grafted Hyaluronic Acid System with Enhanced Injectability and Biostability for Photo-Controlled Osteoarthritis Gene Therapy

Zhijie Chen, Feng Zhang, Hongbo Zhang, Liang Cheng, Kaizhe Chen, Jieliang Shen, Jin Qi, Lianfu Deng, Chuan He, Hélder A. Santos*, Wenguo Cui**

Dr. Z. Chen, L. Cheng, K. Cheng, J. Shen, Prof. J. Qi, Prof. L. Deng, Prof. C. He, Prof. W. Cui
Department of Orthopaedics, Shanghai Key Laboratory for Prevention and Treatment of Bone and Joint Diseases, Shanghai Institute of Traumatology and Orthopaedics
Ruijin Hospital
Shanghai Jiao Tong University School of Medicine
197 Ruijin 2nd Road, Shanghai 200025, P. R. China
E-mail: drhechuan@sina.com; wgcui80@hotmail.com.

F. Zhang, Prof. H.A. Santos
Drug Research Program, Division of Pharmaceutical Chemistry and Technology, Faculty of Pharmacy, University of Helsinki, Helsinki, FI-00014, Finland
Email: helder.santos@helsinki.fi.

Prof. H. Zhang
Pharmaceutical Sciences Laboratory and Turku Bioscience Centre, Åbo Akademi University, FI-20520, Turku, Finland

Prof. H.A. Santos
Helsinki Institute of Life Science (HiLIFE), University of Helsinki, FI-00014, Helsinki, Finland

Keywords: osteoarthritis gene therapy; DNA grafted hyaluronic acid; spherical nucleic acids; enhanced injectability; long-term bio-stability and anti-inflammation

Supplemental Materials and Methods

Table S1. The DNA strands used for the HA-SNAs modification.

Sense DNA	5'-GCTCCGAGATGAATTTTT-3'-N_3
Anti-sense DNA	5'-TTGTTGTTCATCTCGGAGCTTTTT-3'-SH

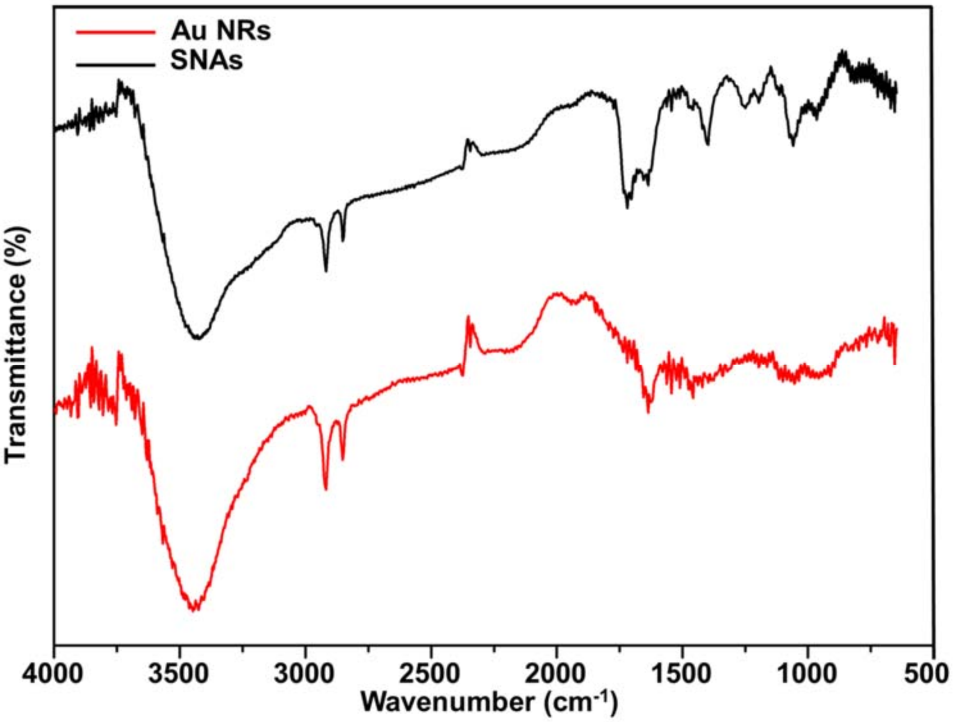

Figure S1. FTIR spectra of the Au NRs and SNAs.

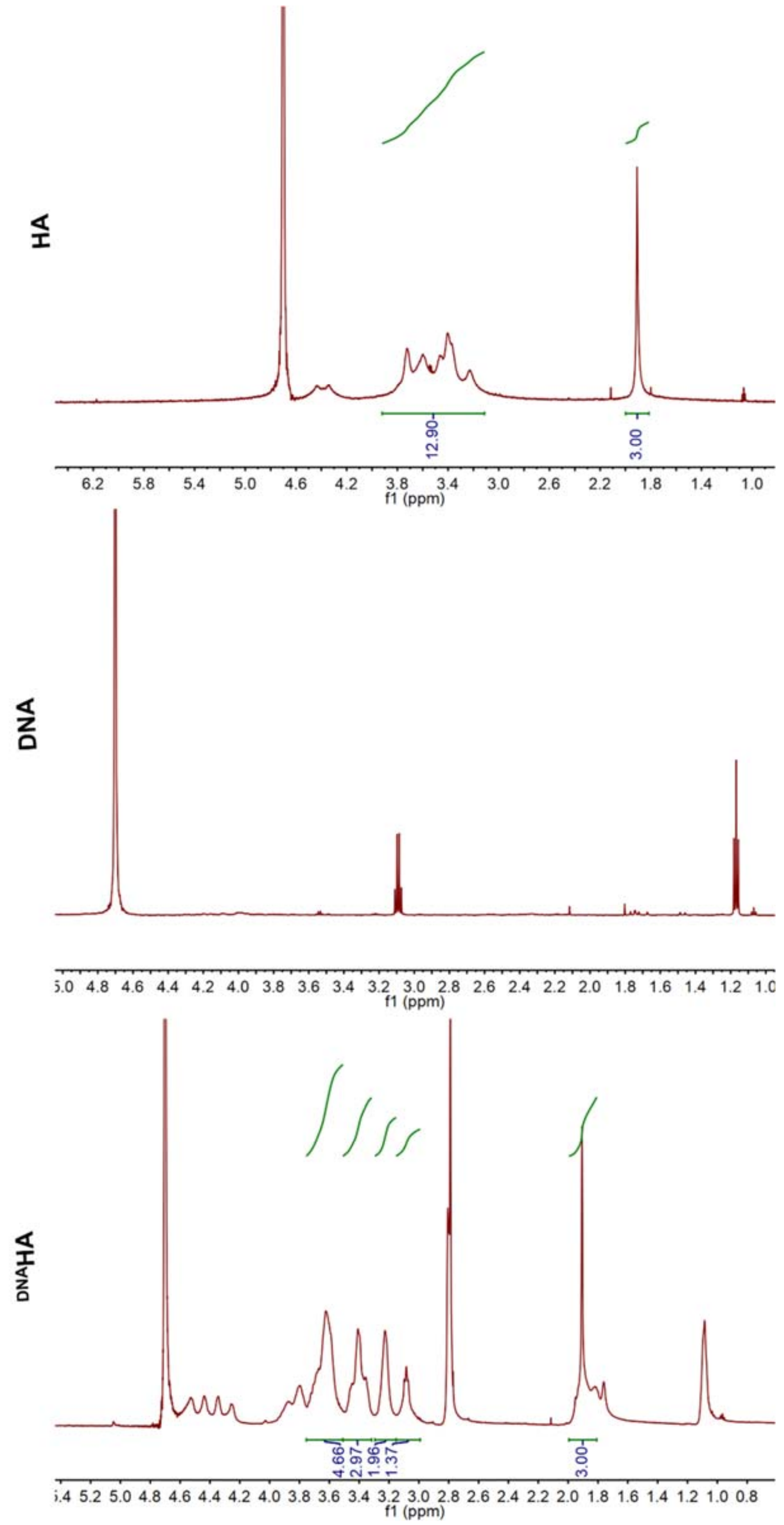

Figure S2. H^1-NMR spectrum of the HA, DNA and ^{DNA}HA.

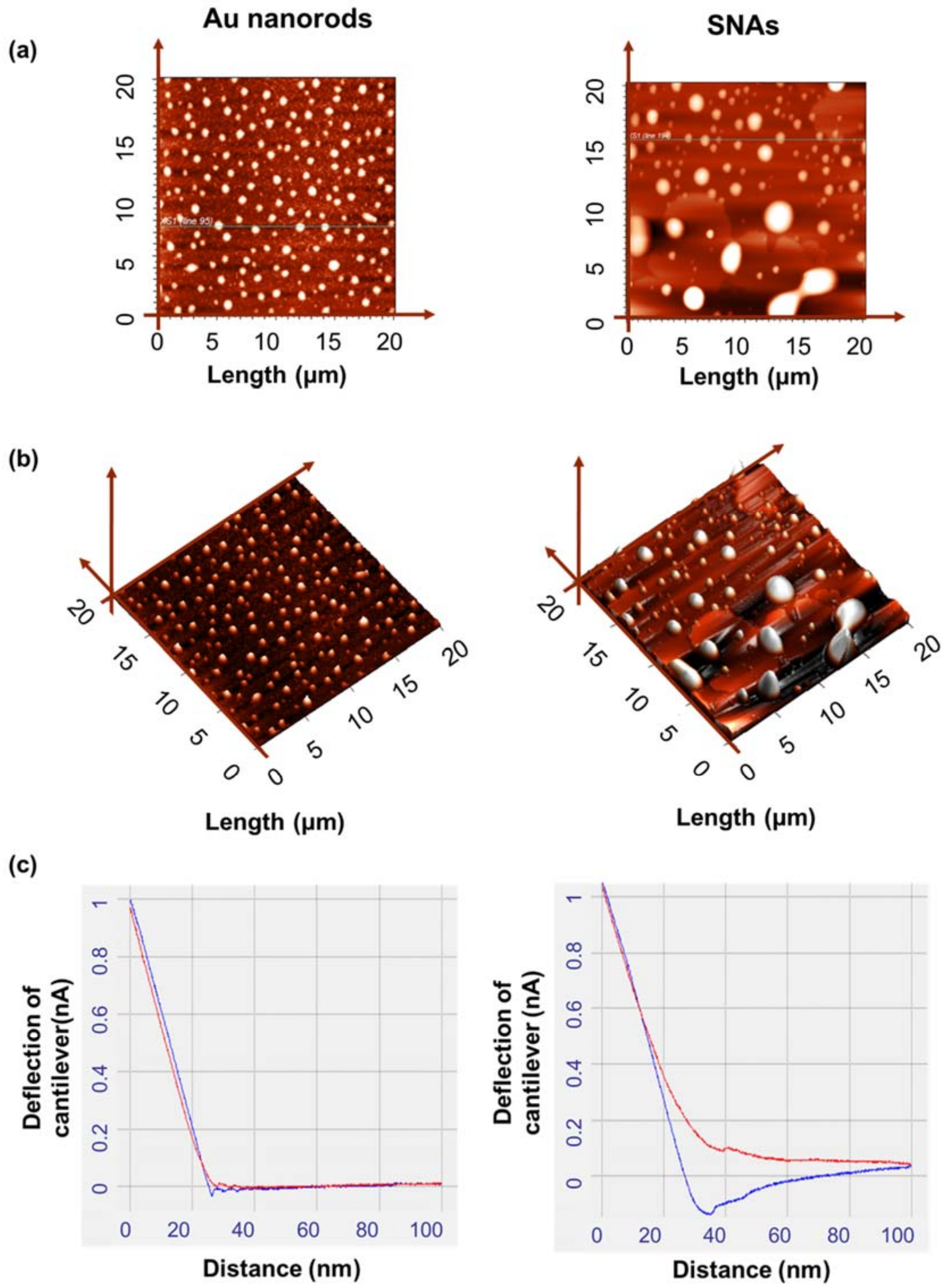

Figure S3. Atomic force microscopy (AFM) and force curve images of Au nanorods and SNAs. (a) 2D-Images and (b) 3D-Images of Au nanorods and SNAs from AFM. While Au nanorods showed similar ultrasmall sizes at about 20−30 nm, the size of SNAs was about 60−70 nm. (c) Force curves showed an enhanced viscous force of SNAs compared with Au nanorods. These results illustrate a successful DNA modification onto the Au nanorods.

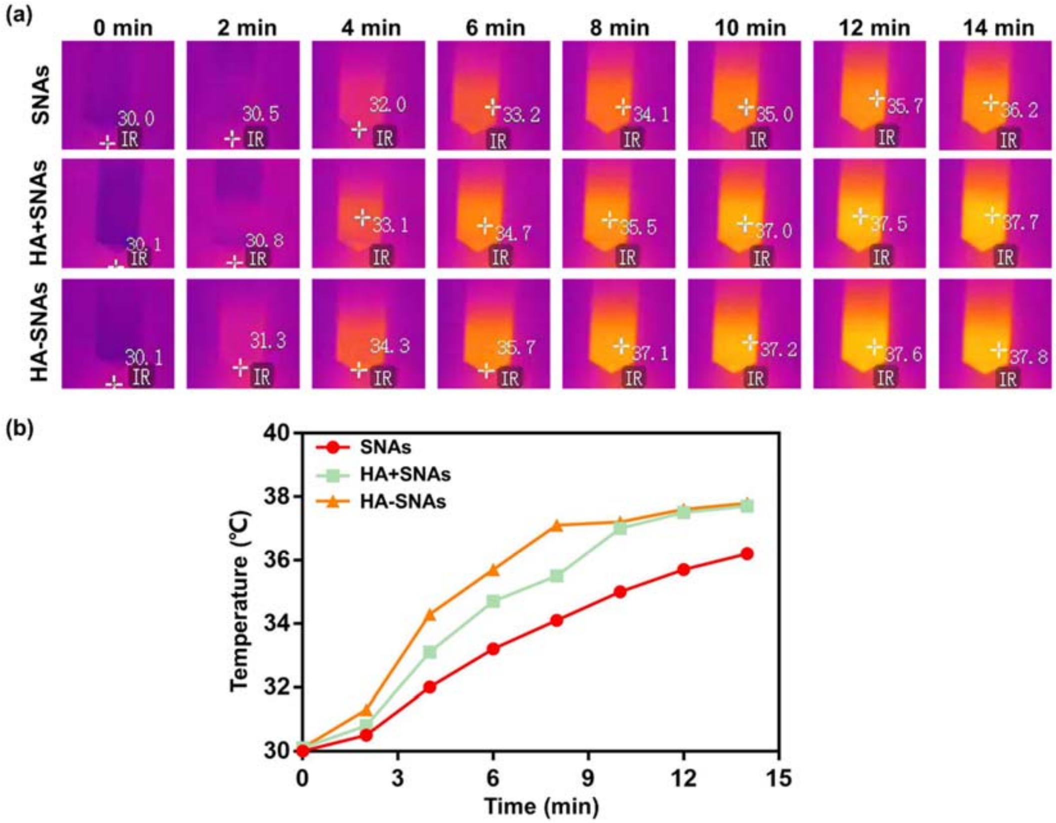

Figure S4. The photothermal images and corresponding temperature profile. (a) The photothermal images of SNAs, HA+SNAs, HA-SNAs irradiated at 808 nm (1.0 W cm^{-2}) for 14 min respectively. (b) The temperature profiles of SNAs, HA+SNAs, HA-SNAs irradiated at 808 nm (1.0 W cm^{-2}) for 14 min, respectively.

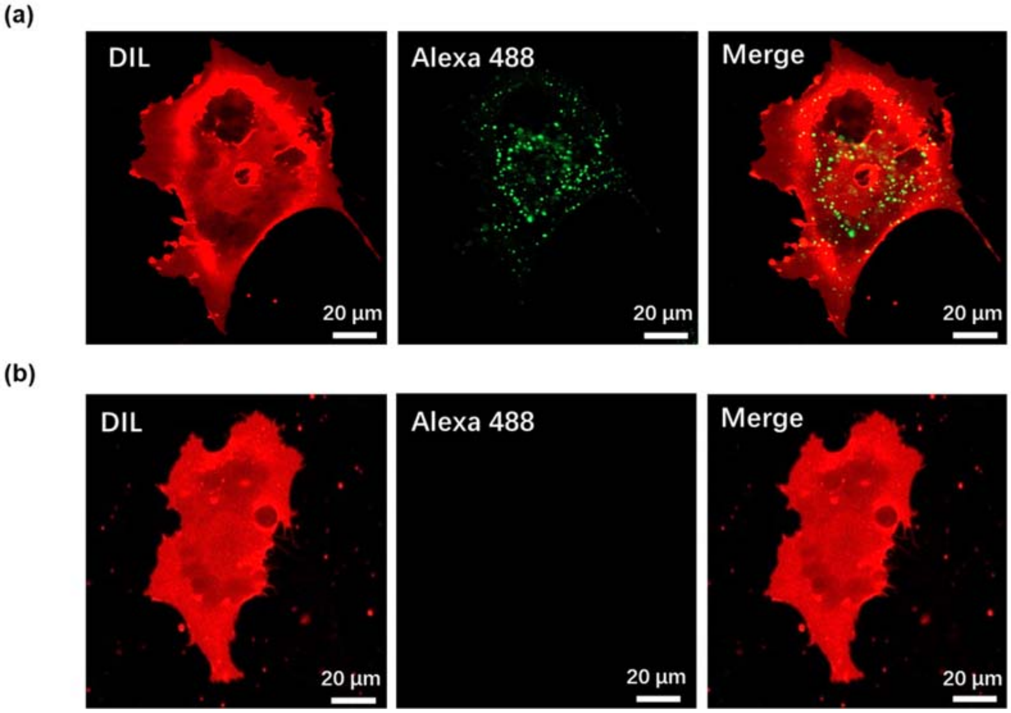

Figure S5. Confocal image of cellular uptake of fluorescence-labelled SNAs in chondrocytes. Alexa 488 labelled SNAs and ^{DNA}HA were incubated with chondrocytes for 12 h, and were observed by confocal microscopy. (a) Alexa 488 labelled SNAs bind to cellular membrane and enter into chondrocytes via endocytosis. (b) Alexa 488 labelled ^{DNA}HA did not enter into chondrocytes. Cell membranes were stained with Dil (red), DNA were labelled with Alexa 488 (green). These results illustrate a greater internalization of SNAs into chondrocytes.

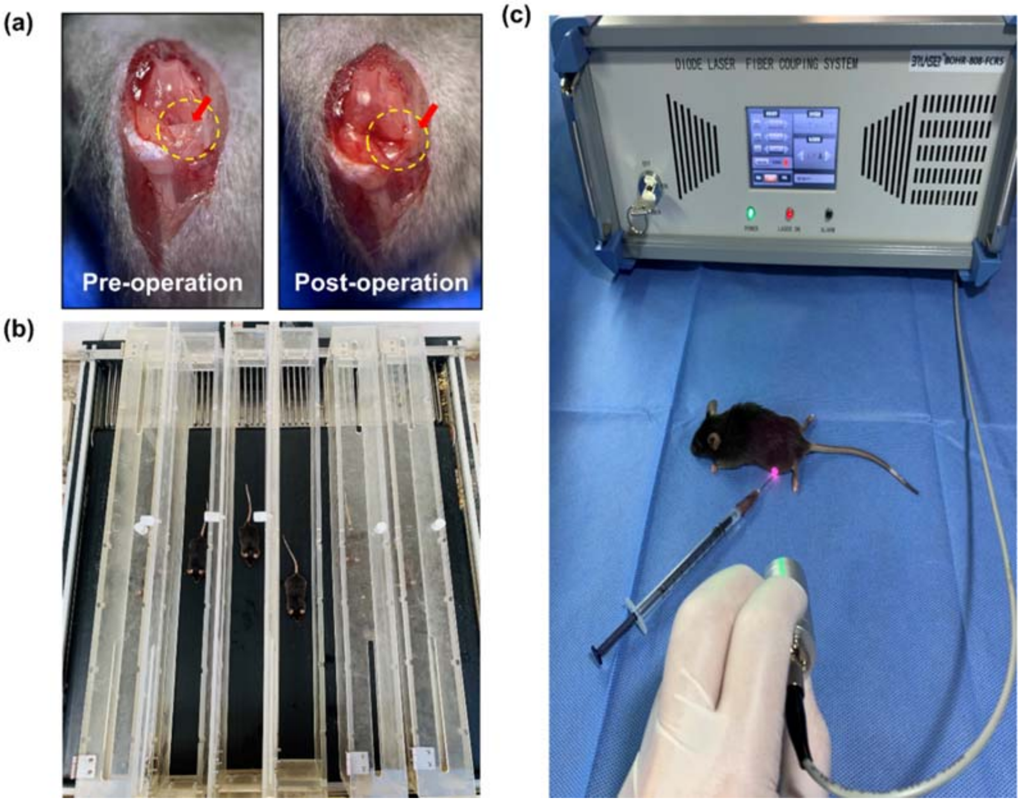

Figure S6. The mouse osteoarthritis model based on DMM surgery and running train and the NIR-light controllable release model after intra-articular injection. (a) Left, exposure of the mediate meniscus (arrow) of the mouse knee joint; Right, the morphology of mouse knee joint after removing the mediate meniscus (arrow). (b) The mice receive a running train on an electronic level treadmill at a speed of 20 m min^{-1} for 1 h every other day to accelerate osteoarthritis of the knee joint. (c) An 808 nm NIR light source (1 W cm^{-2}) was used to irradiate the knee joint for 5 min, then the NIR light source was turned off, and after 1h, the NIR light source was turned on to irradiate for another 5 min, and this cycle lasted for 6 h per day.
